# Autoimmune hematological diseases following haploidentical donor hematopoietic stem cell transplant compared with matched sibling and unrelated donor

**DOI:** 10.18632/oncotarget.15710

**Published:** 2017-02-24

**Authors:** Weiran Lv, Zhiping Fan, Fen Huang, Na Xu, Li Xuan, Guopan Yu, Qianli Jiang, Hongsheng Zhou, Ren Lin, Xin Zhang, Jing Sun, Qifa Liu

**Affiliations:** ^1^ Department of Hematology, Nanfang Hospital, Southern Medical University, Guangzhou 510515 China

**Keywords:** autoimmune hematological diseases, risk factors, treatment, donors, allogeneic hematopoietic stem cell transplantation

## Abstract

Autoimmune hematological diseases (AHDs) occur more frequently than other autoimmune complications after allogeneic hematopoietic stem cell transplantation (allo-HSCT) and are often refractory to treatment. This study was to analyze the incidence and risk factors of AHDs as well as their response to treatment. Four hundred and forty-five adult malignant hematopoietic disorders underwent allo-HSCT were enrolled in this retrospective study, including 124 haploidentical donor (HRD), 140 unrelated donor (MUD) and 181 HLA-matched sibling donor (MSD) transplants. Twelve patients developed AHDs, including 6 autoimmune hemolytic anemia and 6 Evans syndrome. Evans syndrome all occurred in HRD transplants. The 3-year cumulative incidence of AHDs was 4.0 ± 1.3%, and HRD had higher incidence than MUD (8.7 ± 3.0% vs 1.8 ± 1.2%, *P* = 0.012) and MSD (8.7 ± 3.0% vs 3.5 ± 2.6%, *P* = 0.004 ). The steroids combined with Cyclosporine A were acted as the first line treatment, and the response rate was 73%. No patients experienced recurrence at a median follow up of 313 days after stopping treatment. HRD transplants (vs MUD: HR, 5.87; CI, 1.24 to 27.73; *^p^* = 0.026 and vs MSD: HR, 7.70; CI, 1.63 to 36.44; *P* = 0.010) and concurrent chronic graft versus host disease (HR, 3.76; CI, 1.18 to 11.92; *P* = 0.025) were risk factors for AHDs.

## INTRODUCTION

Autoimmune hematological diseases (AHDs) occur more frequently than other autoimmune complications after allogeneic hematopoietic stem cell transplantation (allo-HSCT) [1–7]. AHDs may involve a single lineage of blood cells, such as autoimmune hemolytic anemia (AIHA), immune thrombocytopenia (ITP) or 2 and/or 3 lineages such as Evans syndrome. Data on incidence of AHDs has been the object of several studies in a large cohort, with estimates of the incidence are between 2% to 6% patients, and the most prevalent AHDs is AIHA [2–10]. The optimal therapeutic approach of AHDs is no consensus. The steroids are usually the first line treatment despite knowing that AHDs are often refractory to this traditional therapy [2, 5, 9, 11–14]. The treatment of steroid refractory patients is not yet well established. In this sense, the different single and combined immunosuppressive treatments such as cyclosporine A(CsA),methotrexate, and fluorouracil (CFM), cyclophosphamide(CTX), rituximab, mycophenolate mofetil (MMF) and pentostatin are used [1, 15–19]. The existing data about AHDs are mainly obtained from HLA-matched sibling donor (MSD) and unrelated donor (MUD) transplants including umbilical cord blood. Recently, with technical advances in human leukocyte antigen (HLA) typing, graft versus host disease (GVHD) prophylaxis and supportive care, alternative donors such as haploidentical donor (HRD) have been widely used [20, 21]. However, few studies reported the outcome of AHDs in HRD transplants in large groups of patients.

To better understand the incidence, risk factors and prognosis, and management of AHDs, we carried out a retrospective analysis of 445 adult malignant hematopoietic disorders underwent allo-HSCT at our single-centre, including 124 cases underwent HRD, 140 cases MUD and 181 cases MSD transplants.

## RESULTS

Patient and transplant characteristics are shown in Table 1. Of the 445 patients, 124 received HRD, 140 MUD and 181 MSD transplants. There were significantly different among the 3 groups in the category of patient age (*P* = 0.001), sex (*P* = 0.008), disease status at transplants(*P* = 0.016), source of stem cells (*P* < 0.001), HLA disparity (*P* < 0.001), ATG/CD25 used (*P* < 0.001), PLT engraftment time (*P* = 0.001), WBC engraftment time (*P* < 0.001) and CMV viremia (*P* < 0.001). All patients achieved hematopoietic reconstitution except for 6 patients who died of infections and 3 patients who died of regimen-related toxicity within 2 weeks post-transplantation. In addition, analyses of chimerism showed that all patients achieved full donor chimerism by day +30 post-transplantation except for 8 patients who died of graft rejection and infection.

**Table 1:** Patient and transplant characteristics

Characteristics	HRD	MUD	MSD	*P*
**No. of patients**	124	140	181	
**Sex M/F**	90/34	81/59	101/80	0.008
**Median age, y (range)**	26 (14–57)	28 (14–50)	32 (14–59)	0.01
Primary diseases mye/lym/MDS	68/52/4	59/64/7	88/79/13	NS
**Disease status at HSCT** CR/non-CR	90/34	101/39	152/29	0.016
**Source of stem cells** PBSC/ PBSC+BM	7/117	140/0	172/9	< 0.001
**HLA disparity** matched/ mismatched	4/120	101/39	181/0	< 0.001
**ABO matched** Y/N	71/53	58/82	91/90	0.035
**Sex matched** Y/N	67/57	71/67	77/104	NS
**ATG/CD25 used&^#x203B;^** Y/N	110/14	138/2	11/170	< 0.001
**TBI used** Y/N	77/47	88/52	99/82	NS
**Median MNCs, 10^8^/kg (range)**	7.90 (3.09–13.32)	7.54 (2.76–12.87)	7.3 (3.43–13.47)	NS
**Median CD34+ count, 10^6^/kg (range)**	6.6 (1.64–13)	7.05 (1.2–20.80)	6.24 (0.67–18.26)	NS
**PLT engraftment, d(range)**	15 (9–90)	13 (9–49)	12 (9–100)	0.001
**WBC engraftment, d(range)**	13 (9–70)	12 (9–40)	11 (8–47)	< 0.001
**CMV viremia** Y/N	96/28	80/60	78/103	< 0.001
**Acute GVHD** Y/N	72/52	73/67	93/88	NS
**Chronic GVHD** Y/N	25/99	20/120	34/147	NS

Abbreviations: M, male; F, female; mye, myelogenous diseases, including AML, AUL and CML; lym, lymphoid diseases, including ALL and NHL; CR, complete response; PBSC, peripheral blood stem cell; BM, bone marrow; TBI, total body irradiation; MNC, mononuclear cell; PLT: platelet; WBC: white blood cell; CMV, cytomegalovirus; Y, Yes; N, No.

*ATG (antithymocyte globulin) was given either as prophylaxis or for treatment of GVHD.

### Incidence and characteristics of AHDs

In the retrospective study, AIHA and Evans syndrome only were documented. Of the 445 patients, 12 patients developed AHDs, including 6 AIHA (2 cases in HRD, 2 cases in MUD and 2 case in MSD) and 6 Evans syndrome (in HRD cases ). The overall 3-year incidence of AHDs post-transplantation was 4.0 ± 1.3%, including 8.7 ± 3.0%, 1.8 ± 1.2% and 3.5 ± 2.6%, respectively, in HRD, MUD and MSD transplants (*P* = 0.005). It was higher in these patients undergoing HRD compared with those undergoing MUD (*P* = 0.012 for HRD vs MUD) and MSD transplants (*P* = 0.004 for HRD vs MSD), but not different between MUD and MSD transplants(*P* = 1.000). The median time of AHDs onset post-transplantation was 196 days (60 to 756 days) post-transplants, including 164, 233 and 565 days, respectively, in HRD, MUD and MSD (*P* = 0.004). At the time of AHDs onset, 5 patients were concurrent chronic graft-versus-host disease, and all of the 12 patients receiving immunosuppressive agents, including 7case as GVHD prophylaxis with gradually tapered and 5 as GVHD treatment. The specific immunosuppressive therapies at the AHDs onset are shown in Table 2. In the patient underwent MSD transplants, the onset of AHDs was preceded within 2 weeks by an active viral infective episode (herpes simplex virus). The 12 AHDs patients didn't receive any drugs which could induce hemolytic anemia within a month before the onset of AHDs.

**Table 2:** Transplant and clinical characteristics of patients with AHDs

Case No.	Diagnosis	Sex	Age at HSCT	Type	ABO matched	Time to AHDs	cGVHD / time of cGVHD to AHD (d)	CMV	ATG	Type of antibody	Disease status at HSCT	Immunosuppressive therapies at the onset of AHDs	Treatment	Out-come	Time to response (d)
1	AML	M	46	HRD	No	245	Yes^b^/43	Yes	Yes	IgG	CR	MMF(0.25g 2/d ) +tacrolimus (0.5 mg 2/d)	prednisone+CsA	CR	25
2	MDS	M	21	HRD	No	203	Yes^c^/86	Yes	Yes	IgG	non-CR	MMF(0.25g 2/d ) +CsA (150 mg 1/d) +methylprednisolone (32 mg 1/d)	methylprednisolone+CsA	CR	17
3	T-LBL	M	18	MUD	Yes	312	Nill	Yes	Yes	IgG	non-CR	CsA (50 mg 2/d) +methylprednisolone (32 mg 1/d)	prednisone+CsA	CR	13
4	AML	M	25	HRD	Yes	133	Yes^b^/32	Nill	Yes	IgG	CR	CsA (50 mg 2/d) +methylprednisolone (20 mg 1/d)	prednisone+CsA	CR	24
5	ALL	M	37	HRD	No	189	Nill	Yes	Yes	IgG	non-CR	methylprednisolone (60 mg 1/d)	methylprednisolone	Died^a^	—
6	AUL	M	38	MSD	No	375	Yes^c^/94	Yes	Nill	IgG	non-CR	methylprednisolone (40 mg 1/d) +prograf (1.5 mg 2/d)	CsA+methylprednisolone+rituximab	PR	97
7	ALL	F	29	MSD	Yes	756	Nill	Nill	Yes	IgG	CR	methylprednisolone (32 mg 1/d)	methylprednisolone+CsA	CR	45
8	MDS	F	46	HRD	No	60	Nill	Yes	Nill	IgG	non-CR	MMF (0.5g 2/d) +CsA (50 mg 2/d) +methylprednisolone (40 mg 3/d)	methylprednisolone+CsA	CR	20
9	ALL	F	20	HRD	Yes	140	Nill	Nill	Yes	IgG	CR	MMF(0.25g 1/d ) +CsA (50 mg 2/d) +methylprednisolone (40 mg 1/d)	CsA+methylprednisolone+rituximab	CR	101
10	ALL	M	18	HRD	Yes	104	Nill	Yes	Yes	IgG	CR	MMF (0.2g 2/d) +tacrolimus) 0.5 mg 2/d) +methylprednisolone (40 mg 1/d)	CsA+methylprednisolone+rituximab	PR	89
11	ALL	M	16	MUD	No	154	Yes^c^/39	Yes	Yes	IgG	CR	CsA (50 mg 2/d) +methylprednisolone (32 mg 1/d)	methylprednisolone+CsA	CR	19
12	ALL	M	19	HRD	Yes	203	Nill	Yes	Yes	IgG	CR	MMF (0.5g 2/d) +CsA (50 mg 2/d) +methylprednisolone (40 mg 3/d)	methylprednisolone+CsA	CR	55

Abbreviations: M, male; F, female; Y, yes; N, no; MP, methylprednisolone; P, prednisone; R, rituximab; cGVHD: chronic GVHD.

^a^ This patient died of infectious shock two days after the diagnosis of AHDs.

^b^ Limitation cGVHD.

^c^ Extensive cGVHD.

### Treatment and outcome of AHDs

The patients all received the steroids (2 mg/kg) combined with CSA (keeping the concentration between 200–400 ng/ml) as the first-line of treatment on the basis of original immunosuppressive agents expect for one patient who died of infectious shock at 2 days after the diagnosis of AHDs. (Table 2) After 4weeks’ treatments, 8 cases had response to the initial treatments, including 6 cases CR, and 2 cases PR (the 2 PR cases finally obtained CR after continued first line therapy). The effective rate of initial treatments was 73%, with the median time of CR was 22 days (range,13–55 days). Rituximab was administered in the 3 cases obtaining no response and one patient received CR and 2 PR after rituximab treatment. The overall effective rate was 100%. There was no difference of the efficacy rate in this three types of HSCT (*P* = 0.510). And there was also no difference in response rate between AIHA and Evans syndrome (*P* = 0.886). Recurrence of AHDs was not observed in these cases at a median follow-up of 313 days (range, 153 to 1051) after stopping treatment. In addition, there was no evidence of underlying disease relapse at diagnosis of AHD. The treatment outcomes are showed in Table 2 and Table 3.

**Table 3:** Detailed information about the diagnosis and outcome

Case No.	At diagnosis				After 4 weeks’ treatment			
	WBC (3.5-9.5 G/L)	Hb (115-150g/L)	PLT (125-350G/L)	Coomb test	WBC (3.5-9.5 G/L)	Hb (115-150g/L)	PLT (125-350G/L)	Coomb test
1	3.13	59	40	DAT(+) ;IAT(+)	5.65	121	135	DAT(−) ;IAT(−)
2	1.57	59	36	DAT(+) ;IAT(−)	3.66	117	128	DAT(−) ;IAT(−)
3	1.68	52	123	DAT(+) ;IAT(−)	6.32	142	139	DAT(−) ;IAT(−)
4	1.78	66	41	DAT(+) ;IAT(−)	4.55	124	152	DAT(−) ;IAT(−)
5	3.45	46	101	DAT(+) ;IAT(−)	—	—	—	DAT(−) ;IAT(−)
6	3.49	61	115	DAT(+) ;IAT(−)	4.27	103	143	DAT(−) ;IAT(−)
7	4.08	57	129	DAT(+) ;IAT(−)	4.14	126	131	DAT(−) ;IAT(−)
8	1.32	58	45	DAT(+) ;IAT(−)	4.98	132	157	DAT(−) ;IAT(−)
9	3.47	56	138	DAT(+) ;IAT(−)	4.91	125	129	DAT(−) ;IAT(−)
10	1.96	60	16	DAT(+) ;IAT(−)	3.85	105	109	DAT(+) ;IAT(−)
11	4.21	62	144	DAT(+) ;IAT(−)	3.97	137	146	DAT(−) ;IAT(−)
12	4.34	63	13	DAT(+) ;IAT(−)	4.67	129	142	DAT(−) ;IAT(−)

Abbreviation: Hb, hemoglobin.

### Risk factors for AHDs

**Figure 1:**
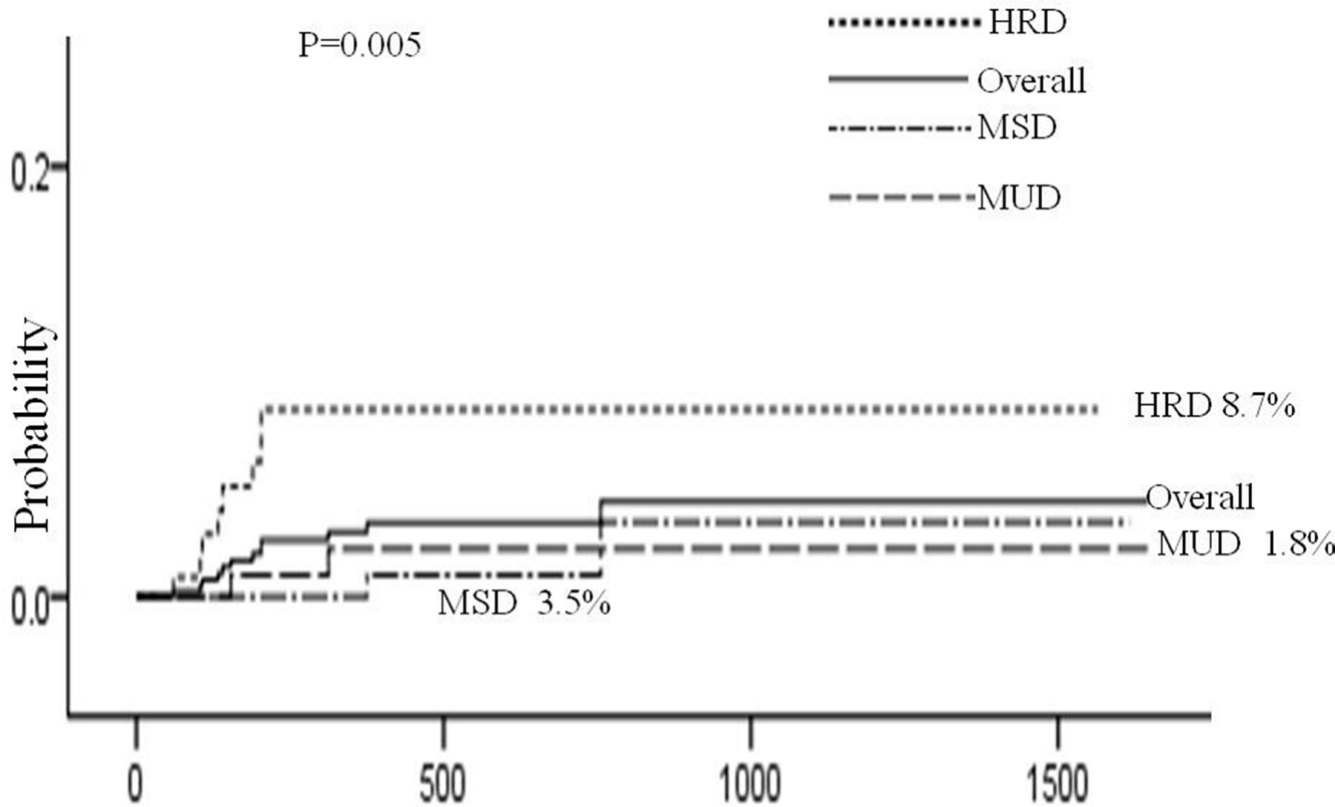
Cumulative incidence of AHDs according to type of donor The 3-year cumulative incidence of AHDs was 4.0 ± 1.3%, and HRD had higher incidence than MUD (8.7 ± 3.0% vs 1.8 ± 1.2%, *P* = 0.012) and MSD (8.7 ± 3.0% vs 3.5 ± 2.6%, *P* = 0.004 ). But there was no difference between MUD and MSD transplants (*P* = 1.000).

**Figure 2:**
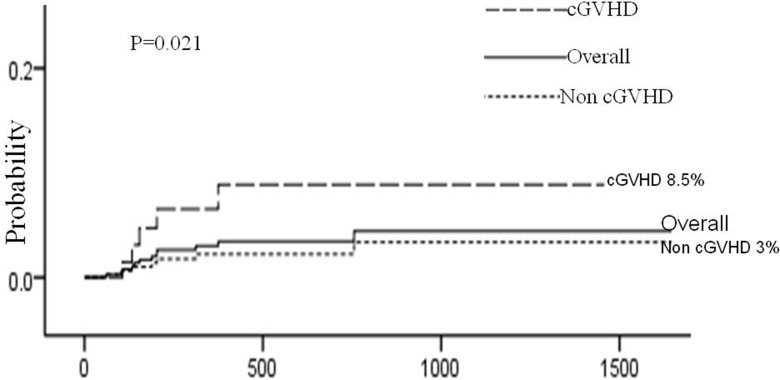
Cumulative incidence of AHDs according to the development of cGVHD The 3-year cumulative incidence of AHDs was 4.0 ± 1.3%, and the patients developed cGVHD had higher incidence than those who did't develope cGVHD (8.5 ± 3.7% vs 3.0 ± 1.3%, *P* = 0.021 ).

Risk factors for AHDs are presented in Table 4. Univariate analysis showed that 4 factors were associated with AHDs: HRD, source of stem cell, HLA disparity and chronic GVHD. Multivariate analysis using Cox regression model showed that HRD (HRD vs MUD: hazard ratio [HR], 5.87; 95% confidence interval [CI], 1.24 to 27.73; *P* = 0.026 and HRD vs MSD: hazard ratio [HR], 7.70; 95% confidence interval [CI], 1.63 to 36.44; *P* = 0.010) and chronic GVHD (hazard ratio [HR], 3.76; 95% confidence interval [CI], 1.18 to 11.92; *P* = 0.025) remained statistically significant. (Figures 1, 2)

**Table 4:** Univariate and multivariate analysis for risk factors of AHDs

Variable	Univariate	Multivariate (RR)
Male vs female	NS	NS
Patient age,> 29 y old, ≤ 29 y old	NS	NS
myelogenous vs lymphoid vs MDS**^#x203B;^**	NS	NS
MSD vs HRD	*P* = 0.017	*P* = 0.010 (7.70) 95% CI:1.627–36.442
HRD vs MUD	*P* = 0.049	*P* = 0.026 (5.87) 95% CI:1.241–27.730
MSD vs MUD	NS	NS
CR vs non-CR	NS	NS
PBSC vs PBSC+BM	*P* = 0.044	NS
HLA matched vs mismatched	*P* = 0.032	NS
ABO matched vs mismatched	NS	NS
Sex matched vs mismatched	NS	NS
ATG/CD25 used vs non-used	NS	NS
TBI used vs non-used	NS	NS
CMV viremia positive vs negative	NS	NS
aGVHD vs non-aGVHD	NS	NS
cGVHD vs non-cGVHD	*P* = 0.044	*P* = 0.025 (3.76) 95% CI:1.184–11.920

Abbreviation: aGVHD: acute GVHD.

^#x203B;^:biphenotypic acute leukemia was excluded.

### Serological analysis of AHDs patients

The 12 patients with AHDs all had positive DAT. The IAT was positive both in serum and eluate in one patient and the other 11 patients had negative IAT. All AHDs patients were warm-reacting (lgG) autoantibodies and they all had clinical and laboratory signs of significant hemolysis. No antibodies against the ABO system were found.

### Lymphocyte recoveries analysis of AHDs patients

We have monitored the lymphocyte recoveries after HSCT, especially at 3 and 6 months. (Table 5) At 3 and 6 months after transplantation, 391 patients (46 died and 8 censored) and 324 patients (101 died and 20 censored), respectively, could be analyzed. The data showed that there was no imbalance between the reconstitution of B and T cells in AHDs patients at 90 and 180 days after transplantation.

**Table 5:** Lymphocyte recoveries at 3 and 6 months after HSCT

Group	3 months			6 months		
	Total lymphocyte counts (10^9^/L)	Proportion of T cells (%)	Proportion of B cells (%)	Total lymphocyte counts (10^9^/L)	Proportion of T cells (%)	Proportion of B cells (%)
AHDs	1.311	67.19	3.20	1.733	64.92	8.02
non-AHDs	1.536	69.74	4.21	1.722	66.09	8.03
*P* value	0.464	0.595	0.783	0.757	0.795	0.516

### Survival

**Figure 3:**
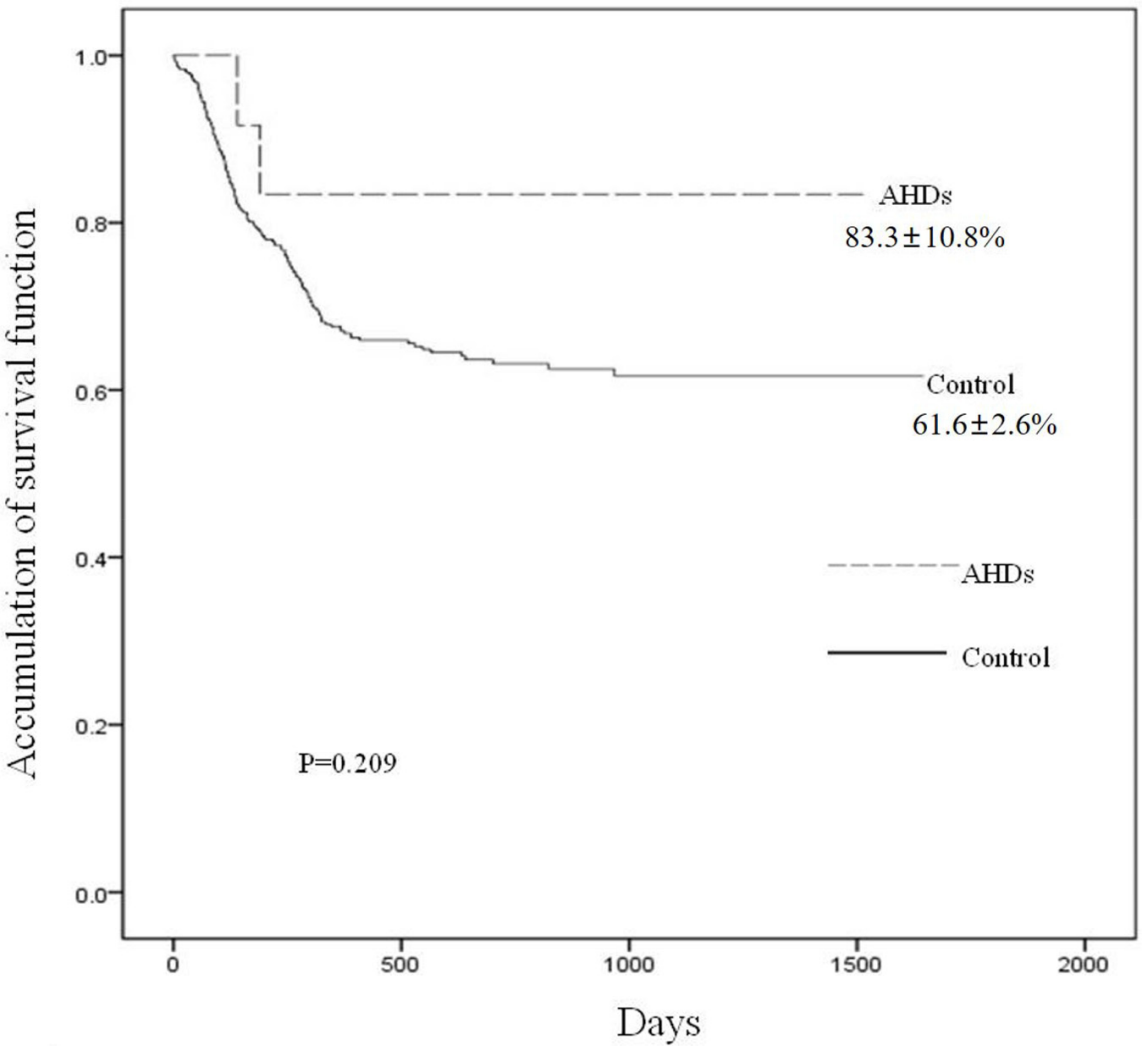
Accumulation of survival function according to the development of AHDs The 3-year overall survival (OS) post-transplants was 62.2 ± 2.6%. Patients with AHDs had not significantly increased risk of mortality compared with patients who received HSCT at the same period (83.3 ± 10.8% vs 61.6 ± 2.6%) (*P* = 0.209).

With a median follow-up of 355.5 days (range, 2–1464 days) post-AHDs, 10 patients were alive and 2 died, including one died of cytomegalovirus pneumonia and the other died of multiple organ failure caused by bacterial infection without detecting specific pathogenic bacterium. The 3-year overall survival (OS) post-transplants was 62.2 ± 2.6%. Patients with AHDs (the 3-year survival was 83.3 ± 10.8%) had not significantly increased risk of mortality compared with patients who received HSCT at the same period (the 3-year survival was 61.6 ± 2.6%) (*P* = 0.209) (Figure 3).

## DISCUSSION

AHDs are a well-recognized complication after allo-HSCT, and the most prevalent AHDs is AIHA. The incidence of AHDs post-transplantation is between 2% to 6%, depending on source of donors, ATG used, recipient age and HLA typing, and so no [6–8, 13, 22]. The incidence of AHDs of MUD transplants is higher than that of MSD transplants, especially umbilical cord blood donor, and the pediatric has a higher incidence than adult [2, 5, 9]. The median time of AHDs onset is between 5 and 12 months post-transplantation [2–7]. In this report, 3-year cumulative incidence of AHDs was 4.0 ± 1.3% and the median time was 196 days, which were consistent with reports [2–8]. And the incidence of AHDs in HRD transplants was higher than that in the MUD and MSD transplants. However, there was no difference between MUD and MSD transplants, which was not consistent with the literatures [2, 3, 9]. One reason might be the small sample in our study. Interestingly, Evans syndrome all occurred in HRD transplants, and 6 of 8 AHDs presented as Evans syndrome in HRD transplants. This was different from the literatures that reported AHDs post-HSCT mainly present as AIHA and rarely present as Evans syndrome in MUD and MSD transplants [3, 23]. As far as we know, this is the first report about the incidence and characteristic of AHDs in HRD transplants in large cohort.

The fundamental mechanisms of AHDs are not clear yet, some researchers suggested that immune dysregulation or incomplete immune reconstitution might be the pathogenetic mechanism leading to the development of these complications [3]. The risk factors of AHDs were mainly associated with source of donors, cGVHD [6, 8], lymphocyte depletion of graft with *ex vivo* lymphodepletion or *in vivo* lymphodepletion using ATG or alemtuzumab [7, 13, 22], younger [9] and HLA typing [13] and peripheral blood stem cells, and so on [2]. Our results showed that HRD and cGVHD were the risk factor of AHDs. The risk of AHDs in HRD transplants was higher than the MUD and MSD transplants. A reasonable interpretation of high risk of AHDs in HRD transplants is that these patients have high incidence of cGVHD [24, 25] and exsited the HLA- mismatch. In addition, there were reports that the onset of AHDs was associated with infections, especially virus infection [3, 6]. In our report, only one of the patients with AHDs was observed had active viral infective episode in 2 weeks preceding the onset of AHDs. Although the patients in our centre had high incidence of CMV viremia, there was no patient that had CMV viremia with a month before the onset of AHDs. What's more, in this report, we surprisingly found that 6 of the 8 AHDs cases presented as Evans syndrome in HRD transplants, whereas the other 4 AHDs cases that occurred in MUD or MSD did not developed Evans syndrome. What brought these on is worthy of study.

The management of AHDs post-transplantation is complex and no consensus. The steroids are usually used as the first line treatment for AHDs, but the effective rate was only about 10%–40% [3, 9, 19, 23]. To those who failed to response to the steroids treatment, the second/third-line treatments was added such as rituximab, CsA, MMF and so on. And the effective rate of these second/third-line treatments was about 60–85% [3, 9, 19, 23]. In our report, we took the steroids combined with CSA as the first line treatment and our response rate was 73% which was significantly higher than the literatures [3, 9, 19, 23]. What's more, there was no difference in the efficiency between AIHA and Evans syndrome. Meanwhile, our results also showed a lower recurrence rate. These good responses whether are related to the combination of drugs is worthy of study. In addition, AHDs whether contributes to increased mortality is not defined yet [9, 14, 23]. Our result suggested that AHDs post-HSCT didn't contribute to increase mortality.

The deficiency of our report was that a retrospective data was analyzed and other autoimmune hematological diseases such as neutropenia and thrombocytopenia were excluded because these diseases were not paid attention in our clinical practice so that we failed to conclude the overall incidence of AHDs and the incidence of autoimmune neutropenia and thrombocytopenia.

In conclusion, our data suggest that the patients undergoing HRD transplants have high incidence of AHDs, and AHDs in HRD transplants mainly presented as Evans syndrome compared with MUD and MSD transplants. Corticosteroids combined with CSA as the first line treatment of AHDs results in good response and low recurrence rate. AHDs don't increase the transplant-related mortality.

## MATERIALS AND METHODS

Between December 2011 to June 2015, 445 patients with adult malignant hematopoietic disorders underwent allo-HSCT were enrolled in this retrospective study, including 278 males and 167 females whose median age was 29 years [range, 14–59]. The primary diseases included acute myeloid leukemia (AML, *n* = 167), acute lymphocytic leukemia (ALL, *n* = 185), acute undifferentiated leukemia (AUL, *n* = 19), chronic myeloid leukemia (CML, *n* = 40) myelodysplastic syndrome (MDS, *n* = 24), non-Hodgkin lymphoma (NHL, *n* = 10). The patients who were enrolled in our study didn't have the history of AHDs or other autoimmune diseases before allo-HSCT. The study protocol was approved by Ethics Committee of Nanfang Hospital, Southern Medical University.

### Transplantation

HLA typing of recipients and donors was carried out by high-resolution molecular techniques. Donor selection was as follows: if a suitable MSD (i.e. a sibling donor matching > 8/10) was available, the donor was chosen. If a suitable MSD was unavailable, a suitably matched MUD was used as the alternative. If a suitable MSD or MUD was unavailable within the timeframe appropriate for the patient's malignancy and clinical circumstances (i.e. patients in high risk achieved complete remission received 3–4 cycles of consolidation therapy; patients in no response (NR) urgently needed allo-HSCT; patients in CR2 or beyond), HRD was administered [26]. The selection of conditioning regimen was based on the primary diseases and disease state at transplants. Usually, BuCY (busulfan+cyclophosphamide) was used for myeloid hematological diseases with complete response (CR), while TBI+CY (Total Body Irradiation+cyclophosphamide) used for lymphoblastic hematological diseases with CR and the intensive conditioning containing TBI was used for patients with noCR at transplants [26, 27]. Three hundred and nineteen patients received peripheral blood stem cell (PBSC) transplants and 126 patients PBSC combined with bone marrow transplants. Cyclosporine A (CsA) combined with methotrexate (MTX) (at day +1, 3, and 6) were used for GVHD prophylaxis in MSD transplants. CsA, MTX, and antithymocyte globulin (ATG) (7.5 mg/kg) were used for GVHD prophylaxis in MUD transplants, and CsA, MTX, and +ATG 7.5–10 mg/kg) + MMF (0.5 g, 2/day × 28 days) in HRD transplants. Corticosteroids were used for the initial treatment of acute GVHD, and ATG or ATG combined with CD25 monoclonal antibody and other immunosuppressants were used in corticosteroid-resistant acute GVHD cases. Corticosteroids and CsA were used initially for the treatment of chronic GVHD, and the combination with many immunosuppressants were used for the treatment of chronic GVHD that failed initial therapy [26]. The transplantation protocols were approved by Ethics Committee of Nanfang Hospital and informed consent was obtained from all patients and donor.

### Serology

ABO, Rh typing were performed on donor and recipient samples before transplantation on DiaMed gel columns. The direct antiglobulin test (DAT) was performed in all patients with clinical suspicion of hemolytic anemia on DiaMed gel columns. Indirect antiglobulin test(IAT) was performed to screen antibody in serum and eluate. The presence of IgG and C3d on red cells was demonstrated by testing with monospecific reagents at 37°C.

### The diagnosis and treatment of AHDs

Diagnosis of AIHA and Evans syndrome was according to lectures. [6, 15, 23]. Criteria of AIHA included: (1) positive DAT, (2) positive indirect antiglobulin test (IAT) with broad reactivity to RBC in serum and eluate, (3) clinical and laboratory evidence of hemolysis (increase of LDH and bilirubin levels, decrease of hemoglobin and haptoglobin levels or increase in transfusion requirements), (4) Differentiation diagnosis: Cases of DAT positivity due to ABO antibodies, as well as those with history of AIHA or positive DAT before HSCT, were excluded. Patients who never had DAT were presumed not to have clinically significant AIHA. Patients with positive DAT but evidence of nonimmune hemolysis, eg,microangiopathic hemolytic anemia, were also excluded. Furthermore, the diagnosis of AHDs following allogeneic hematopoietic stem cell transplantation should excluded primary and secondary poor graft function. Diagnostic criteria of Evans syndrome: Defined by combination (either simultaneously or sequentially) of ITP and AIHA with a positive DAT. AHDs treatments are based on our medication guide. Steroids combined with CsA were acted as first-line of treatment for AHDs, and four weeks was a cycle of treatment. The second-line drugs, including rituximab, CTX and many other kinds of drugs, were used for patients with AHDs who failed to respond to the first-line therapy. Criteria of effectiveness: (1) Complete remission (CR): Hb level of 12 g/dL or more in the absence of any transfusion without features of hemolysis (normal bilirubin and LDH levels ± normal haptoglobin level if performed). (2). Partial response (PR): Hb level of at least 10 g/dL with an increase of at least 2 g from baseline and a persistent hemolysis. (3). No remission(NR): fail to meet the above two criterias. Refractory AHDs was defined as failure to respond after 4-week first-line treatment.

### Statistical analysis

Patient follow-up was updated on June, 2016. The endpoints included the incidence and risk factors of AHDs as well as its response to treatment. Variables related to patients, disease, transplant characteristics among the 3 groups were compared using the Pearson × 2 test or Fisher's exact test for categorical variables and the 1-way ANOVA for continuous variables. Numerical variables were analyzed as categories based on their values being below or above the median of the entire cohort. The cumulative incidences of AHDs and OS were analyzed with the method of Kaplan–Meyer, comparing the groups using the logrank test (Mantel–Haenszel). Cox proportional hazards regression model were used for analysis of risk factors for time-to-event variables. All *P* values were 2 sided and considered significant if < 0.05. Statistical analyses were performed with SPSS Version 19.0.
